# Cannabis Indica

**Published:** 1885-07

**Authors:** Wm. D. Kempton


### Cannabis Indica.

BY WM. D. KEMPTON, D.D.S.

Read before the Mad River Dental Society.

Since the last meeting of the Mississippi Valley Association of Dental Surgeons I have been experimenting with this agent in extracting teeth. I had Mr. Wm. Karrmann prepare a fluid extract, each ounce of which represents the active principle of an ounce of the herb. I was somewhat skeptical in regard to its efficacy as I had had so many failures with other much-lauded remedies, but after using it in over 150 cases, I am satisfied that very often it enables the operator to extract teeth "*without pain*," and with very little pain in nearly all cases.

Whilst I do not pretend to explain its modus operandi, yet I have observed that when applied to the gums it produces a peculiarly anæmic appearance in from five to twenty minutes. It is then, and *then only*, that anæsthesia is present. In from three to five per cent. of cases, however, this effect is not produced, and consequently there is no anæsthesia.

To apply it I made a clamp of brass wire, on the principle of dressing forceps that open when pressed, with a loop on the end of each beak, in which a pledget of cotton is drawn and saturated with the agent. This is allowed to remain in contact with the gum for five minutes, is then removed and a few drops added, the apparatus replaced, and so on till the desired effect is produced. The patient should hold his head over the spittoon to allow the surplus agent and the copious flow of saliva to escape.

